# Next-Generation Sequencing Techniques to Diagnose Culture-Negative Subacute Native Aortic Endocarditis

**DOI:** 10.3201/eid3107.241739

**Published:** 2025-07

**Authors:** Delphine Vetterli, Morgana Zennaro, Virginie Tacchini, Joannes Alexander Lobrinus, Virginie Prendki, Vladimir Lazarevic, Jacques Schrenzel

**Affiliations:** Geneva University Hospitals Department of Medicine, Geneva, Switzerland (D. Vetterli, M. Zennaro); Geneva University Hospitals Division of Cardiology, Geneva (V. Tacchini); Geneva University Hospitals Department of Pathology, Geneva (J.A. Lobrinus); Geneva University Hospitals Department of Rehabilitation and Geriatrics, Geneva (V. Prendki); Genomic Research Laboratory, Faculty of Medicine, Geneva University, Geneva (V. Lazarevic, J. Schrenzel); Geneva University Hospitals Division of Infectious Diseases, Geneva (V. Prendki, J. Schrenzel); Geneva University Hospitals Bacteriology Laboratory, Geneva (J. Schrenzel)

**Keywords:** endocarditis, bacteria, streptococci, infective endocarditis, sequence analysis, cell-free nucleic acid, DNA, bacteremia, mcfDNA, Switzerland

## Abstract

Next-generation sequencing might improve diagnosis of infective endocarditis. A case in Switzerland was initially attributed to *Solobacterium moorei* bacteria. Metagenomic analysis of the affected heart valve detected *Streptococcus gordonii*, but not *S. moorei*, illustrating that the results of molecular detection can vary depending on sampling time and anatomic site.

Plasma microbial cell-free DNA (mcfDNA) refers to extracellular microbial DNA in plasma, which has a half-life of a few minutes (*1*). Next-generation sequencing using mcfDNA is emerging as a diagnostic tool in infections with negative cultures, including endocarditis. Persistence of mcfDNA is associated with metastatic infection (*2*). We used next-generation mcfDNA sequencing to identify the causative agent in a fatal case of infective endocarditis.

An 89-year-old man with aortic stenosis and preserved heart function sought care for weakness in Geneva, Switzerland. He had recently sought care at University Hospital of the Canary Islands (Tenerife, Spain) after a fall; elevated troponin (2,172 ng/L) and procalcitonin (157 mg/L) were observed. He received empiric meropenem and linezolid before he returned to Switzerland against medical advice. In Geneva, he had no fever or peripheral signs of endocarditis. Investigations revealed mild inflammation (C-reactive protein 17 mg/L), acute kidney injury (creatinine 387 µmol/L), stroke, and carotid stenosis. Transesophageal echocardiography showed an aortic valve perforation and a 4 mm para-aortic abscess at the root of the aorta, without suspected vegetation (Figure 1). Blood cultures remained negative after 5 days. We started conservative treatment with ceftriaxone (2 g every 12 h intravenously) and vancomycin (15 mg/kg every 12 h intravenously). mcfDNA next-generation sequencing (Noscendo, https://noscendo.com) identified *Solobacterium moorei* (13 reads). We adjusted the patient’s treatment to ceftriaxone (2 g every 12 h intravenously) and metronidazole (500 mg every 6 h intravenously) for 6 weeks. 

**Figure 1:**
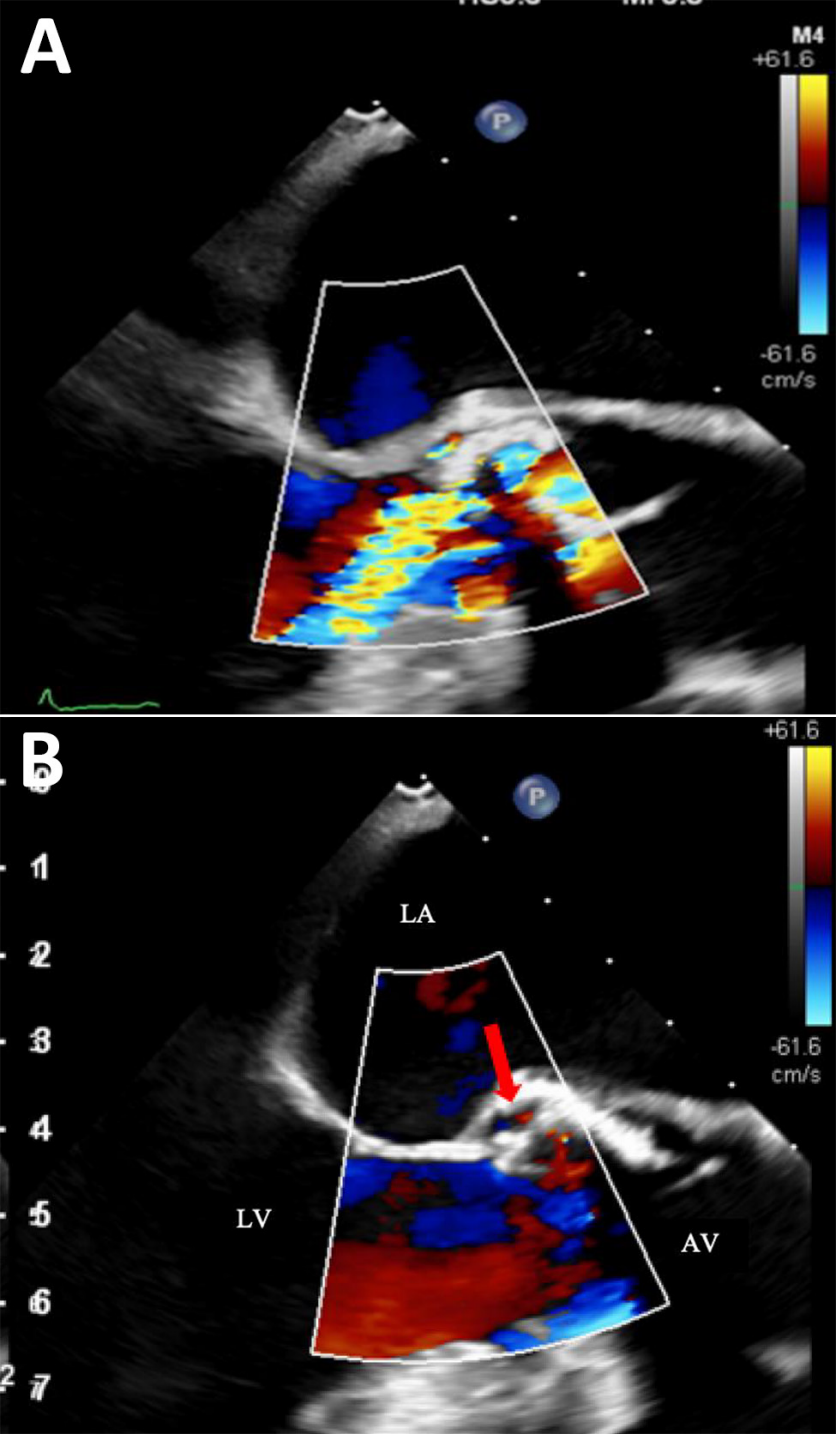
Color doppler echocardiography images from fatal case of subacute native aortic endocarditis, Geneva, Switzerland. A) Mid-esophageal long-axis view during diastole, showing moderate to severe aortic regurgitation. B) Mid-esophageal long-axis view with left ventricular chamber, aortic valve, and aortic root during systole. Red arrow indicates systolic flow with the pseudo-aneurysm. AV, open aortic valve; LA, left atrial; LV, left ventricle.

Despite initial improvement, the patient experienced heart failure and a second-degree AV block. His condition declined 12 weeks later, and he died. At autopsy, the heart showed a heavily calcified, perforated noncoronary leaflet of the aortic valve with a 15 × 10-mm blood-filled neocavity beneath it, extending to the valvular ring (Figure 2). Although we did not detect pus, our findings strongly suggested infective endocarditis because degenerative processes do not typically cause valve perforation or cavity formation. Those conditions are consistent with infective endocarditis (IE). A second mcfDNA test detected no bacteria.

**Figure 2:**
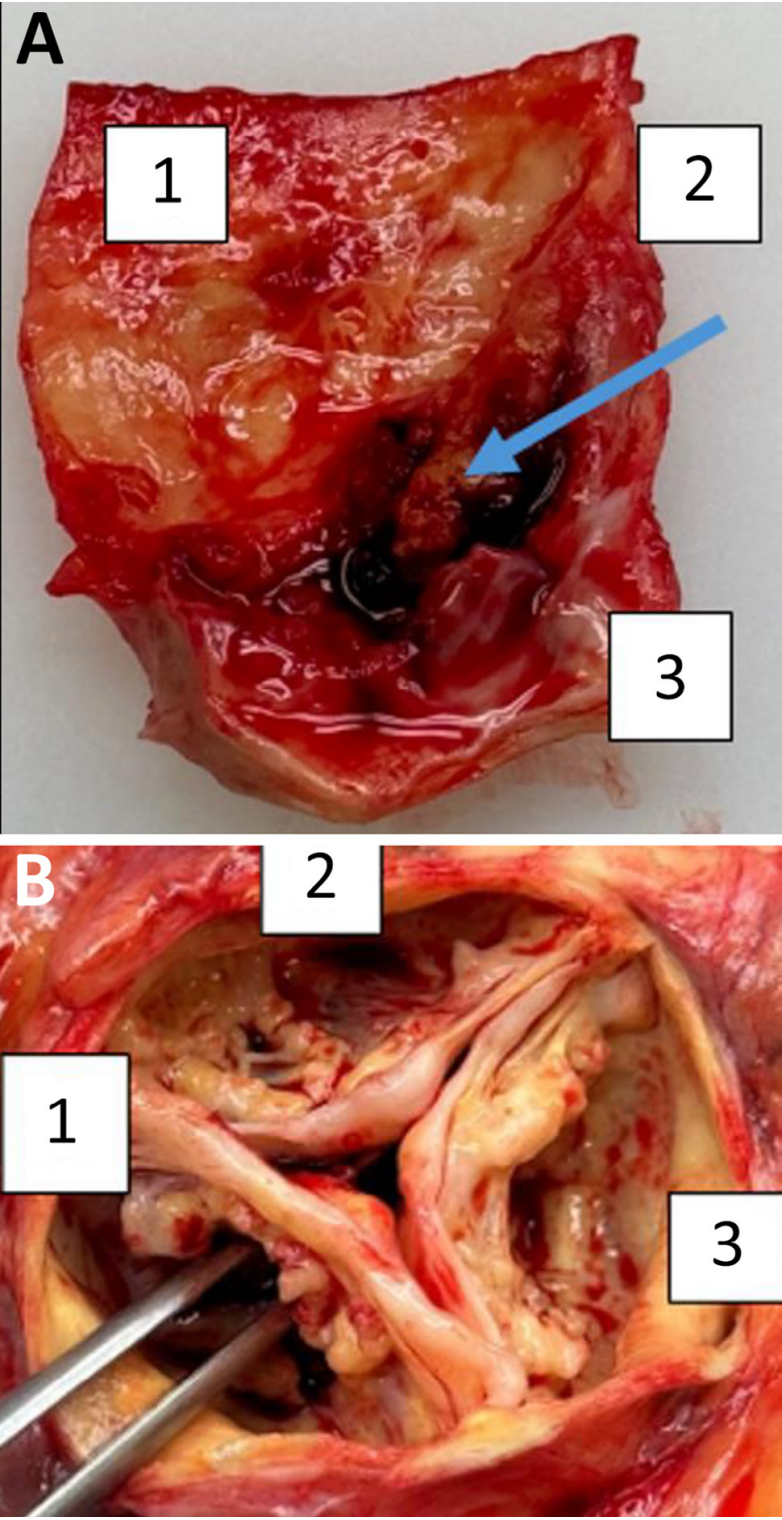
Autopsy results from fatal case of subacute native aortic endocarditis, Geneva, Switzerland. A) Autopsy material of the ascendant aorta (1) with the open blood-filled neocavity of 15 × 10 mm (blue arrow) just beneath the perforated noncoronary leaflet of the AV (2) and extending to the valvular ring (3). B) Autopsy material of the open aortic valve with perforated noncoronary leaflet (1), left coronary leaflet (2), and right coronary leaflet (3).

We performed metagenomic next-generation sequencing (mNGS) of the valve tissue as previously described (*3*). We identified 1.28 million human reads and 36,629 reads from the spiked (8.5 × 10^4^) control organism *Bacillus spizizenii*, along with 4,654 reads from *Streptococcus gordonii*, 146 reads from *S. sanguinis*, and 11 reads from *Cutibacterium acnes* (European Nucleotide Archive accession no. PRJEB81450). We used MetaPhlAn2 (https://huttenhower.sph.harvard.edu/metaphlan2) to confirm *S. gordonii*, which suggested it was the dominant pathogen in tissue (*4*). Reads identified as *S. sanguinis* were likely *S. gordonii* as well because of their high genomic similarity. We detected no *Solobacterium moorei* in the tissue. *S. moorei* is a gram-positive anaerobic rod from oral and intestinal microbiota. Although rarely detected, it has been implicated in human infections, especially in immunocompromised patients (*5*–*7*). Its identification is difficult because of its slow growth. It is generally susceptible to antimicrobial drugs for anaerobic infections, although resistance to rifampin and moxifloxacin has been reported (*8*)*.*

This case demonstrates the utility of mcfDNA and metagenomic sequencing in culture-negative endocarditis. After negative routine work-up, we performed mcfDNA because the conservative management prevented valve resection. Although *S. moorei* was detected in plasma initially, a follow-up mcfDNA test 6 weeks after antimicrobial treatment was negative. That result likely indicates bacterial clearance, because it slightly exceeds the median 38-day positivity duration observed in infective endocarditis (*9*). *S. gordonii* was the only pathogen identified in valve tissue. The discrepancy between cfDNA and mNGS may reflect differing bacterial loads, sampling timing, or antimicrobial impact (*10*).

Our findings suggest an endocarditis caused by both *S. gordonii* and *S. moorei* organisms in which *S. moorei* mcfDNA predominated during the initial sampling but its culture likely failed because of antimicrobial exposure. In contrast, *S. gordonii* DNA seemed to persist longer in the valve tissue, suggesting greater stability in that environment. The absence of *S. moorei* in the valve tissue raises questions about its pathogenic role; its presence in mcfDNA could represent a transient bacteremia, another unrelated site of infection, or a contamination, but its relative abundance may have masked initial detection of *S. gordonii* bacteria. *S. gordonii* is a known endocarditis pathogen causing destructive IE, and its pathogenic role is therefore highly probable.

In case of a high suspicion of IE and when surgery is not feasible, we advise collecting additional blood or plasma samples at least 2 times within the first 24–48 hours. If blood cultures yield negative results, stored samples can undergo mcfDNA analysis. Testing multiple samples improves diagnostic reliability by minimizing the risk for unrelated transient bacteremia or contamination. If valve removal occurs, mNGS should be done as a final test for pathogen identification.

In summary, we report a case of destructive native aortic valve endocarditis without fever or marked inflammation. mcfDNA and mNGS were essential to identify the pathogen. Molecular diagnostics are valuable in culture-negative infections, particularly when conventional methods and tissue sampling are limited.
